# Prospective Investigation of Video Game Use in Children and Subsequent Conduct Disorder and Depression Using Data from the Avon Longitudinal Study of Parents and Children

**DOI:** 10.1371/journal.pone.0147732

**Published:** 2016-01-28

**Authors:** Peter J. Etchells, Suzanne H. Gage, Adam D. Rutherford, Marcus R. Munafò

**Affiliations:** 1 School of Society, Enterprise and Environment, Bath Spa University, Newton St Loe, Bath, United Kingdom; 2 School of Experimental Psychology, University of Bristol, 12a Priory Road, Bristol, United Kingdom; 3 MRC Integrative Epidemiology Unit at the University of Bristol, 12a Priory Road, Bristol, United Kingdom; 4 UK Centre for Tobacco and Alcohol Studies, University of Bristol, 12a Priory Road, Bristol, United Kingdom; 5 UCL Division of Biosciences—Genetics, Evolution and Environment, University College London, Gower Street, London, United Kingdom; Johns Hopkins Bloomberg School of Public Health, UNITED STATES

## Abstract

There is increasing public and scientific concern regarding the long-term behavioural effects of video game use in children, but currently little consensus as to the nature of any such relationships. We investigated the relationship between video game use in children, degree of violence in games, and measures of depression and a 6-level banded measure of conduct disorder. Data from the Avon Longitudinal Study of Parents and Children were used. A 3-level measure of game use at age 8/9 years was developed, taking into account degree of violence based on game genre. Associations with conduct disorder and depression, measured at age 15, were investigated using ordinal logistic regression, adjusted for a number of potential confounders. Shoot-em-up games were associated with conduct disorder bands, and with a binary measure of conduct disorder, although the strength of evidence for these associations was weak. A sensitivity analysis comparing those who play competitive games to those who play shoot-em-ups found weak evidence supporting the hypothesis that it is violence rather than competitiveness that is associated with conduct disorder. However this analysis was underpowered, and we cannot rule out the possibility that increasing levels of competition in games may be just as likely to account for the observed associations as violent content. Overall game exposure as indicated by number of games in a household was not related to conduct disorder, nor was any association found between shoot-em-up video game use and depression.

## Introduction

Despite frequent expressions of concern in the media, the behavioural and psychological effects of prolonged video game playing are not well understood. Although it has been claimed recently that there is a consensus among video game researchers [1], the evidence for this statement is not convincing [2,3]. Furthermore, in 2013, 230 scholars [4] wrote to the American Psychological Association expressing concerns that policy statements on media violence would be untenable, as they would be based on a literature that shows considerable inconsistency regarding the nature of suggested links between violent media and antisocial or aggressive behaviour [5–12].

One issue is the lack of consistent and robust methodologies across different studies [13]; in particular, few longitudinal studies looking at the relationship between the prolonged use of video games and adolescent social behaviour have been conducted. A recent three-year longitudinal study of game violence and aggression in children aged 10–14 years, which included measures of personality, family attachment, peer delinquency, family violence and depression, suggested that game violence was not associated with any negative behavioural outcomes, and did not predict youth aggression [14]. Others have suggested that violent game use is associated with subsequent development of aggressive behaviour [15], although the magnitude of these associations is small.

Aside from differences in the range and type of potential confounding factors that are adjusted for across such studies, others have made the more general observation that it is difficult to isolate the violent content of video games from other features. For example, after systematically analysing games for levels of violence (and matching for factors such as level of difficulty and pace of action), Adachi and Willoughby [5] found experimentally that more competitive games resulted in more aggressive short-term behaviours. Similarly, a 2013 longitudinal study [16] of nearly 1,500 teenagers concluded that playing competitive games predicted higher levels of aggression over a period of 4 years, after adjusting for violent content. There is therefore a clear need to further assess the relative contributions of competitiveness and violent content in games, and the impact these have on negative behavioural outcomes.

One theoretical framework that has been developed in an attempt to explain some of these findings is the Catalyst Model [17]. In this model, the role of internalised motivations and biological factors is emphasised, as well as ‘catalysing’ effects of environmental factors. Potentially aggressive personalities develop through initial biological dispositions, and environmental stress factors (e.g., a negative family environment) may make a person more likely to engage in aggressive behaviours, and more attracted to playing violent games. Under this model, and unlike other models proposed such as the General Aggression Model [18], violent game use does not cause negative behavioural outcomes. Instead, while aggressive behaviours may be modeled on behaviours seen in games, they would manifest in other ways had the individual not been playing a video game. The same stress factors may also precipitate poor mental health outcomes more generally, given that aggression has been linked to depressive symptoms [19].

It remains unclear how adolescent social behaviour and mental health is affected by video game use. Many studies tend to look at the effects of games generally, while in reality there are a wide range of differences in game style, content and play that may confer differing, even opposite effects on behaviour. We were therefore interested in whether the level of violent content in games is associated with negative behaviour outcomes. We investigated the association between video game use at age 8/9 years, and subsequent measures of conduct disorder and depression at age 15 years, taking into account a wide range of potential confounding factors. We also attempted to assess any relationship between the degree of violence in the games played, and any behavioural outcomes.

## Methods

### Participants

The Avon Longitudinal Study of Parents and Children (ALSPAC) is a prospective, population-based birth cohort study that recruited 14,541 pregnant women living in Avon, UK, with expected delivery dates between 1^st^ April 1991 to 31^st^ December 1992. The cohort has been described in detail previously [20]. The current study is based on the 5,400 young people who completed the Development and Well-Being Assessment (DAWBA) semi-structured interview at the ALSPAC clinic at 15 years of age (4,745 responded to conduct questions and 5,369 to depression questions).

### Ethics Statement

Ethics approval for the study was obtained from the ALSPAC Ethics and Law Committee and the Local Research Ethics Committees. Please note that the study website contains details of all the data available through a fully-searchable data dictionary (http://www.bris.ac.uk/alspac/researchers/data-access/data-dictionary/).

### Measures

#### Video game use

Video game use at age 8/9 years was measured via children’s self-report questionnaire. Type of video game played was assessed with the question: “Which types of computer games do you have at home?” Respondents could answer yes to as many as applicable. Options presented in the questionnaire were: “shoot-em-up”, “sport”, “racing (e.g., Micromachines, Midtown Madness)”, “role-playing (e.g., Dungeons and Dragons)”, “puzzles (e.g., Bubble Bobble)”, “strategy (e.g., Command and Conquer)”, “flight simulator”, “platform (e.g., Sonic)”, “other (e.g., educational or learning games)”.

Responses were used to derive a 3-level measure intended to capture whether or not participants played video games and, if they did, the degree of violence in the games played: 0) does not play video games; 1) plays puzzle games; 2) plays “shoot-‘em-up” games. If participants played games in more than one category, they were categorised upwards. Number of video games was also assessed: participants were asked “How many computer games do you have at home?” and could respond 0, 1–2, 3–4, 5–9 or 10 or more. A further binary measure was created of those who used only racing or sports games (competitive games), and those who reported using only shoot-em-up games (violent games).

### Conduct Disorder and Depression

Conduct disorder and depression were assessed at age 15 via the DAWBA semi-structured interview. Conduct disorder was assessed via parental report and depression via child self-report. From participants’ responses, DAWBA “bands” can be created, corresponding to ordered categorical measures of likelihood of conduct disorder and depression. These are created using computer algorithms, and have been validated in samples of children in the United Kingdom. The bands contain 6 levels, and are based on the probability of disorder, ranging from very unlikely to probable. As well as the ordered categorical variables, the top two levels in each category can be used to create binary “disorder” variables [21]. The DAWBA has been validated in clinical and community samples, and is a useful assessment for large-scale data collection such as that occurring in cohort studies. DAWBA band scores for parental-rated conduct disorder and depression at age 7 were used for exclusion, and adjustment at baseline was used to attempt to minimise the possibility of reverse causation.

### Potential Confounders

Potential confounders, for which there was a theoretical or empirical basis for considering that they might influence any observed association between video game use and subsequent mental health, were: a) pre-birth confounders, including family history of mental health problems (binary measures assessed via maternal questionnaire); maternal education (a 5-level categorical variable assessed via maternal questionnaire); ancestry (assessed via maternal questionnaire); religiosity (assessed as “belief in a divine power” via maternal questionnaire); family structure during pregnancy (assessed via maternal questionnaire); and offspring sex; and, b) childhood confounders, including IQ at age 8 (assessed via Wechsler Intelligence Scale for Children [22]); whether child was a victim of bullying at age 8 (a binary measure assessed via parent and child report); peer problems, emotional problems, and conduct problems at age (assessed via the Strengths and Difficulties Questionnaire [23], analyses of conduct disorder did not adjust for conduct problems, and analyses of depression did not adjust for emotional problems). When we substituted maternal socioeconomic position (a 6-level categorical variable assessed vie maternal questionnaire) for maternal education our results were not substantially altered (results available on request).

### Statistical Analysis

We assessed the relationship between video game use and DAWBA ordered categorical bands of conduct disorder and depression using ordinal logistic regression, before and after adjustment for confounders. We excluded pre-existing conduct problems or depression measured at baseline, in order to assess incidence of the outcome, and adjusted for variation in baseline conduct disorder or depression in those who remained. We confirmed the proportional odds/parallel regression assumption had not been violated using the Brant test [24]. The relationship between video game use and DAWBA binary conduct disorder and depression variables was assessed using logistic regression, before and after adjustment for confounders. We examined the impact of grouped confounders on the association between video game use and conduct disorder and depression by comparing unadjusted estimates (model 1) with those adjusting for pre-birth confounders (model 2), and those further adjusted for childhood confounders (model 3). Relationships of these potential confounders with video game use were assessed via polychoric correlations (available on request). Analyses were carried out in STATA version 12 (Stata Corp LP, College Station TX, USA).

We also conducted multiple imputation of 100 datasets in order to investigate and account for potential bias introduced due to attrition. Over 50 auxiliary variables were used to make the missing-at-random assumption plausible, including measures related to pre-birth and childhood factors. The main analyses were repeated using imputed exposure and confounder data (imputed sample N = 4,745 for conduct outcomes and 5,369 for depression outcomes). These imputed datasets also accounted for pre-existing conduct problems or depression, as appropriate.

## Results

### Characteristics of Participants

Of 14,701 children in the initial full cohort, 5,400 completed at least some of the DAWBA at age 15. From this sample, 2,453 provided video game usage data at age 8/9. The final sample available for analysis consisted of 1,815 children for whom data on the above confounders were available. Of these, a total of 26 participants met the criteria for conduct disorder case status, which increased slightly by video game exposure category (none: 1 / 0.7%; puzzle games: 11 / 1.2%; shoot-em-ups: 14 / 1.6%). A total of 22 participants met the criteria for depression case status, which decreased slightly by video game exposure category (none: 2 / 2.5%; puzzle games: 10 / 1.0%; shoot-em-ups: 8 / 0.9%). The characteristics of all participants, grouped by video game exposure category, are presented in Table 1. For the analyses investigating conduct disorder, 11 participants were excluded from the analysis as they had pre-existing conduct problems, or missing data on that question at age 8/9. For analyses investigating depression, 8 were excluded for pre-existing depression at baseline.

**Table 1:** Descriptive statistics for confounders and outcomes by exposure categories (N = 1,815).

		None (n = 141)	Puzzle games (n = 858)	Shoot-em-ups (n = 816)
**Sex**				
**Female**	n (%)	90 (63.8)	539 (62.8)	274 (33.6)
**Male**	n (%)	51 (36.2)	319 (37.2)	542 (66.4)
**Family history of depression**				
**No**	n (%)	103 (73.1)	633 (73.8)	584 (71.6)
**Yes**	n (%)	38 (26.9)	225 (26.2)	232 (28.4)
**Family history of schizophrenia**				
**No**	n (%)	139 (98.6)	853 (99.4)	809 (99.1)
**Yes**	n (%)	2 (1.4)	5 (0.6)	7 (0.9)
**Maternal education level**				
**CSE**	n (%)	20 (14.2)	55 (6.4)	70 (8.6)
**Vocational**	n (%)	11 (7.8)	65 (7.6)	54 (6.6)
**O level**	n (%)	54 (38.3)	275 (32.1)	295 (36.2)
**A level**	n (%)	37 (26.2)	267 (31.1)	231 (28.3)
**Degree**	n (%)	19 (13.5)	196 (22.8)	166 (20.3)
**Partner during pregnancy**				
**Husband**	n (%)	108 (76.6)	739 (86.1)	701 (85.9)
**Other male**	n (%)	30 (21.3)	106 (12.4)	109 (13.4)
**None**	n (%)	3 (231)	10 (1.2)	4 (0.5)
**Other**	n (%)	0 (0)	3 (0.3)	2 (0.2)
**Religion**				
**No Religion**	n (%)	67 (47.5)	385 (44.9)	360 (44.1)
**Religious**	n (%)	74 (52.5)	473 (55.1)	456 (55.9)
**SDQ peer problem score**	M (SD)	1.28 (1.32)	1.20 (1.47)	1.19 (1.43)
**WISC Total score**	M (SD)	104.7 (16.7)	111.2 (16.2)	108.8 (15.7)

SDQ: Strengths and Difficulties Questionnaire; WISC: Wechsler Intelligence Scale for Children.

### Conduct Disorder

Ordered regression of conduct disorder band (6 levels) at age 15 and genre of game played (none, puzzle, shoot-em-up) at age 8/9 years indicated weak evidence of an association in the unadjusted model (OR = 1.16, 95% CI 0.99 to 1.37, p = 0.067) which was not altered substantially in the partially adjusted (OR = 1.15, 95% CI 0.98 to 1.37, p = 0.095) or fully adjusted (OR = 1.19, 95% CI 1.00 to 1.42, p = 0.050) models (Table 2). That is, in the fully adjusted model, there was a 19% increase in risk of being in a higher conduct disorder band per categorical change in genre of game played at age 8/9 years, although statistical evidence of an association was weak. When conduct disorder case status was used as the outcome, substantially larger odds ratios were seen (as would be expected when using a binary rather than categorical outcome), with an association in the unadjusted model (OR = 1.90, 95% CI 1.04 to 3.48, p = 0.037) which strengthened slightly in the partially adjusted (OR = 1.98, 95% CI 1.06 to 3.69, p = 0.031) and attenuated again in the fully adjusted (OR = 1.87, 95% CI 1.02 to 3.45, p = 0.044) models (Table 2).

**Table 2:** Ordered regression of conduct disorder band (6 levels), conduct disorder (case status) and depression band (6 levels) at age 15 and genre of game played (3 levels) at age 8/9 years excluding those with severe pre-existing outcome symptoms (conduct N = 1,804, depression N = 2,498) and further adjusting for pre-existing outcome symptoms in those remaining.

	Conduct Disorder (6 levels)			Conduct Disorder (case status)			Depression (6 levels)		
Model	OR	95% CI	p-value	OR	95% CI	p-value	OR	95% CI	p-value
**Unadjusted**	1.16	0.99 to 1.37	0.067	1.90	1.04 to 3.48	0.037	0.84	0.73 to 0.97	0.016
**Partially adjusted** †	1.15	0.98 to 1.37	0.095	1.98	1.06 to 3.69	0.031	0.98	0.85 to 1.14	0.808
**Fully adjusted** ‡	1.19	1.00 to 1.42	0.050	1.87	1.02 to 3.45	0.044	0.98	0.85 to 1.14	0.840

^†^ Model adjusted for sex, family history, maternal education level, marital status during pregnancy, and religion.

^‡^ As above, with additional adjustment for experience of bullying, peer problems, emotional adjustment, conduct problems, and Wechsler Intelligence Scale for Children total score at age 8/9 years.

There did not appear to be any association between the *number* of games played at age 8/9 years (which was not stratified by genre) and conduct disorder band at age 15 in the unadjusted, partially adjusted or fully adjusted models. These results are presented in Table 3.

**Table 3:** Ordered regression of conduct disorder band (6 levels) at age 15 and number of games played at age 8/9 years, excluding those with pre-existing conduct problems (N = 2,765).

Model	OR	95% CI	p-value
**Unadjusted**	1.03	0.96 to 1.10	0.412
**Partially adjusted** †	1.02	0.95 to 1.10	0.538
**Fully adjusted** ‡	1.03	0.95 to 1.10	0.470

^†^ Model adjusted for sex, family history, maternal education level, marital status during pregnancy, and religion.

^‡^ As above, with additional adjustment for experience of bullying, peer problems, emotional adjustment, conduct problems, and Wechsler Intelligence Scale for Children total score at age 8/9 years.

### Violent versus Competitive Games

A further sensitivity analysis was conducted whereby those selectively playing shoot-em-up games were compared with those selectively playing competitive racing or sport games. Despite larger effect sizes, there was only weak evidence that shoot-em-ups were associated with an increased risk of conduct disorder in comparison to competitive games (Table 4). The effect size observed increased with adjustment for potential confounders.

**Table 4:** Ordered regression of conduct disorder band (6 levels) at age 15 and competitive versus shoot-em-up games, excluding those with pre-existing conduct problems (N = 1,042), further adjusting for pre-existing CD in those remaining.

Model	OR	95% CI	p-value
**Unadjusted**	1.31	0.83 to 2.08	0.243
**Partially adjusted** †	1.38	0.87 to 2.19	0.172
**Fully adjusted** ‡	1.54	0.94 to 2.50	0.084

Reference group: competitive games (sports/racing)

^†^ Model adjusted for sex, family history, maternal education level, marital status during pregnancy, and religion.

^‡^ As above, with additional adjustment for experience of bullying, peer problems, emotional adjustment, conduct problems, and Wechsler Intelligence Scale for Children total score at age 8/9 years.

### Depression

In order to test the specificity of any associations with mental health outcomes, we also looked at whether there was an association between video game use and depression. Ordered regression of genre of game played at age 8/9 years on depression band (6 levels) at age 15 indicated some evidence of a negative association in the unadjusted model (OR = 0.84, 95% CI 0.73 to 0.97, p = 0.016), but after partial adjustment results attenuated to the null (partially adjusted OR = 0.98, 95% CI 0.85 to 1.14, p = 0.808; fully adjusted OR = 0.98, 95% CI 0.85 to 1.14, p = 0.840) (Table 2).

The analyses of the multiply imputed datasets were of a smaller magnitude than the complete case findings for conduct disorder outcome, but were not markedly different. Findings for depression were very similar to the complete case (Table 5).

**Table 5:** Ordered regression of conduct disorder band (6 levels) and depression band (6 levels) at age 15 and genre of game played (3 levels) at age 8/9 years excluding those with pre-existing outcome in 100 multiply imputed datasets (conduct disorder N = 4745, depression N = 5369.

	Conduct Disorder (6 levels)			Depression (6 levels)		
Model	OR	95% CI	p-value	OR	95% CI	p-value
**Unadjusted**	1.13	0.98 to 1.30	0.083	0.85	0.76 to 0.95	0.005
**Partially adjusted** †	1.10	0.95 to 1.27	0.208	0.98	0.87 to 1.11	0.726
**Fully adjusted** ‡	1.11	0.95 to 1.29	0.193	0.98	0.87 to 1.11	0.471

^†^ Model adjusted for sex, family history, maternal education level, marital status during pregnancy, and religion.

^‡^ As above, with additional adjustment for experience of bullying, peer problems, emotional adjustment, conduct problems, and Wechsler Intelligence Scale for Children total score at age 8/9 years

## Discussion

Our results indicate that playing video games that are more likely to include violent content (i.e., shoot-em-ups) in childhood is weakly associated with an increased risk of conduct disorder in late adolescence. There was also weak evidence that individuals who selectively play shoot-em-ups differed in risk to those who selectively play competitive games. However, the absolute risk of developing conduct disorder is small, and the modest effect sizes we observed should be interpreted in this context. Overall game exposure, as indicated by number of games in a household, was not related to conduct disorder, nor was any association found between video game use and depression.

While our results are broadly in line with findings suggesting that violent game content is associated with increased aggressive tendencies, the associations we observe (and statistical evidence for these) are modest, and do not seem to be consistent with claims that the effects of playing violent video games on aggressive behaviour are of a sizeable magnitude. For example, some have claimed [25] that the magnitude of this effect is larger than the effect of exposure to smoke at work on lung cancer rates; our findings do not support such claims. Although attrition could have biased our complete case findings, analyses using multiple imputed datasets as an attempt to account for this support the complete case analyses.

It is worth considering alternative explanations for our findings. One possibility is unmeasured confounding; for example, Ferguson and colleagues [19] show that adjusting for pre-existing emotional, family and social problems removes the apparent association between violent video game use and aggression. Although we adjusted for potential confounders, we cannot be certain that we fully accounted for residual confounding. Another possibility is reverse causation; Przybylski and colleagues [11] suggest that individual differences in levels of dispositional aggression may influence preference for violent game use. Early-onset versus adolescent-onset conduct disorder have been suggested as different subtypes of conduct disorder, with different aetiologies [26]. We removed those identified as having conduct disorder at baseline, and further adjusted for conduct disorder DAWBA band at age 7 in those who remained, but reverse causation remains a possible explanation for our findings.

Overall, our data are consistent with the Catalyst Model. Although there is a weak association between playing shoot-em-ups at a young age and subsequent negative behaviours, the effect is small and the statistical evidence for these associations is weak. Moreover, there was only very weak evidence that individuals who selectively played shoot-em-up games (which we considered in our analysis as the more violent genre) differed in risk from those individuals who selectively played competitive games, suggesting that violent video game content alone may not be a sufficient indicator of risk for later aggressive behaviour. Furthermore, given that in the present study we do not consider the impact of media exposure aside from video games, an assumption that any association between violent game use and aggression is causal [27] is inappropriate here.

It is also important to consider the nature of the questions asked in the study. Children aged 8 or 9 years old may not know what a ‘shoot-em -up’ game is, so may have not correctly classified games that they were playing. Participants were given exemplar games for some categories, but not all. Nevertheless, given that there was no ‘first/third person shooter’ category, it is reasonable to assume that any games in this genre may have been included in the ‘shoot-em-up’ category. Although video games carry age classifications that should restrict access to inappropriate games, these games may nevertheless be present in the home due to the presence of older siblings, or parents that play them. However, if it is the case that children aged 8–9 are not playing extremely violent games, one possible interpretation of our results is that they may actually underestimate the association of playing shoot-em-ups with later aggressive behavioural outcomes. It would be interesting to explore this association at a later age (e.g., 15 or 16 years), when children have more potential access to other types of games that may contain more violent content.

We attempted to differentiate between types of video game played, and one difference between the game categorisations is the potential amount of violent content within each genre–shoot-em-ups are more likely to contain violence than puzzle games or sports games. Nevertheless, our categorisation of violence may not fully reflect the true nature of the violence in these games. This is a general limitation of all current video game research, and is important because violent content and play styles can vary considerably between games that are broadly categorised as ‘violent’. Moreover, the time point at which participants took part in our study means any games they reported playing are now over 15 years old, which should be taken into consideration when using these results in the context of the potential effects of more recent games. As such, our categories may not adequately represent levels of violence in modern games and do not take into account the amount of time played (although it is worth noting that a recent study [28] showed that the amount of time spent playing games did not have any effect on conduct problems).

The ways in which video games are now being played has changed considerably since these data were collected, especially with the advent of more social methods of play (e.g., cooperative, smartphone-based apps). Therefore our data may not be representative of gaming today. Moreover, modern video games blur traditional genre boundaries in increasingly complex ways–for example, puzzle games (ostensibly a ‘non-violent’ category) now range from simple numerical tasks to more graphic crime scene investigations. Given the nature of the data, we were unable to assess the specific games that individuals played. Therefore, there is still a need to look at how the specific content within individual games may impact upon behaviour. Given that there are differences in the literature in outcomes depending on the tested game content, we believe that it is essential that future research pays closer attention to both the specific content within video games, and also the context in which they are played, and moves away from a generalised discussion of ‘video game’ use.

We also attempted to determine whether factors other than violent game content may be associated with aggressive behaviours. Previous studies [5,29] have shown that when competitiveness is matched in violent and non-violent games, violent content alone is not sufficient to affect aggressive behavioural outcomes. Our analysis suggested that there was weak evidence that those individuals who selectively played shoot-em-up games differed in risk of conduct disorder compared to individuals who selectively played competitive sports and racing games (i.e., those who did not report playing shoot-em-ups). While we were unable to determine the relative strength of association of competitive game styles and violent content to aggressive behaviours, at the very least our results highlight the need for greater caution in studies which consider the impact of violent content in isolation from other potentially important factors. Specifically, we were unable to rule out the possibility that competitiveness, rather than violence, may underlie the associations we observe (assuming that they are indeed causal). In our study, it may well be the case that increasing levels of competition in the games that our participants played may be just as likely to account for the associations we observed. Future research should therefore explore the relationships between both competitiveness and violent content on subsequent behaviour.

In conclusion, our data show a weak association between playing shoot-em-up games at an early age, and an increased risk of conduct disorder in late adolescence. Additionally, our data show weak evidence that those who selectively played shoot-em-ups differed in risk to those who selectively played competitive games; however, our analysis was not sufficiently powered to be able to answer with any certainty whether or not the level of competition present in games accounts for the associations we observed. Larger studies, ideally with richer data on video game use, are required needed to address this question. It is also important to note that most of our sample played shoot-em-up games when they were aged 8, and importantly, only very few met the criteria for conduct disorder case status at age 15. Therefore, the association between violent game use and conduct disorder in fact appears to be weak. We observed no association between violent game use and subsequent depression in adolescence, a result that supports earlier studies [30]. Further studies should more closely examine child, family and situational characteristics that may contribute to any observed behavioural problems. Given this, we caution that our results do not have any necessary policy implications.

## References

[1] Bushman BJ, Gollwitzer M, Cruz C. There is broad consensus: Media researchers agree that violent media increase aggression in children, and pediatricians and parents concur. Psychol Pop Media Culture 2015; 4(3): 200–214. 10.1037/ppm0000046

[2] Etchells PJ, Chambers CD. Violent video games research: consensus or confusion? The Guardian. 10 10 2014 Available: http://www.theguardian.com/science/head-quarters/2014/oct/10/violent-video-games-research-consensus-or-confusion. Accessed: 15 June 2015.

[3] Ivory JD, Markey PM, Elson M, Colwell J, Ferguson CJ, Griffiths MD, et al Manufacturing consensus in a diverse field of scholarly opinions: A comment on Bushman, Gollwitzer, and Cruz (2015). Psychol Pop Media Culture 2015; 4(3): 222–229. 10.1037/ppm0000056

[4] Consortium of Scholars. Scholars’ Open Statement to the APA Task Force on Violent Media 2013. Available: http://www.christopherjferguson.com/APA%20Task%20Force%20Comment1.pdf.

[5] Adachi PJC, Willoughby T. The effect of video game competition and violence on aggressive behaviour: Which characteristic has the greatest influence? Psychol Viol 2011; 1: 259–274. 10.1037/a0024908

[6] Anderson CA, Shibuya A, Ihori N, Swing EL, Bushman BJ, Sakamoto A, et al Violent video game effects on aggression, empathy, and prosocial behavior in eastern and western countries: a meta-analytic review. Psychol Bull 2010; 136(2): 151–173. 10.1037/a0018251 20192553

[7] Ewoldsen DR, Eno CA, Okdie BM, Velez JA, Guadagno RE, DeCoster J. Effect of playing violent video games cooperatively or competitively on subsequent cooperative behavior. Cyberpsychol Behav 2012; 15(5): 277–280. 10.1089/cyber.2011.0308 22489544

[8] Ferguson CJ, Kilburn J. The public health risks of media violence: a meta-analytic review. J Pediatr 2009; 154(5): 759–763. 10.1016/j.jpeds.2008.11.033 19230901

[9] Greitemeyer T, Osswald S. Prosocial video games reduce aggressive cognitions. J Exp Soc Psychol 2009; 45(4): 896–900. 10.1016/J.Jesp.2009.04.005

[10] Hasan Y, Begue L, Bushman BJ. Viewing the world through "blood-red tinted glasses": The hostile expectation bias mediates the link between violent video game exposure and aggression. J Exp Soc Psychol 2012; 48(4): 953–956.

[11] Przybylski AK, Ryan RM, Rigby CS. The motivating role of violence in video games. Pers Soc Psychol B 2009; 35(2): 243–259. 10.1177/0146167208327216 19141627

[12] Strasburger VC. Go ahead punk, make my day: It's time for pediatricians to take action against media violence. Pediatr 2007; 119(6): E1398–E1399. 10.1542/Peds.2007-0083 17545366

[13] Ferguson CJ. Blazing angels or resident evil? Can violent video games be a force for good? Rev Gen Psychol 2010; 14(2): 68–81. 10.1037/A0018941

[14] Ferguson CJ, San Miguel C, Garza A, Jerabeck JM. A longitudinal test of video game violence influences on dating and aggression: a 3-year longitudinal study of adolescents. J Psychiat Res 2012; 46(2): 141–146. 10.1016/j.jpsychires.2011.10.014 22099867

[15] Willoughby T, Adachi PJ, Good M. A longitudinal study of the association between violent video game play and aggression among adolescents. Dev Psychol 2012; 48(4): 1044–1057. 10.1037/a0026046 22040315

[16] Adachi PJ, Willoughby T. Demolishing the competition: the longitudinal link between competitive video games, competitive gambling, and aggression. J Youth Adolescence 2013; 42(7): 1090–1104. 10.1007/s10964-013-9952-2 23595418

[17] Ferguson CJ, Rueda SM, Cruz AM, Ferguson DE, Fritz S, Smith SM. Violent video games and aggression: Causal relationship or byproduct of family violence and intrinsic violence motivation? Crim Justice Behav 2008; 35: 311–332.

[18] Anderson CA, Bushman BJ. Human aggression. Annu Rev Psychol 2002; 53: 27–51. 10.1146/annurev.psych.53.100901.135231 11752478

[19] Ferguson CJ, San Miguel C, Hartley RD. A multivariate analysis of youth violence and aggression: the influence of family, peers, depression, and media violence. J Pediatr 2009; 155(6): 904–908 e903 10.1016/j.jpeds.2009.06.021 19683724

[20] Boyd A, Golding J, Macleod J, Lawlor DA, Fraser A, Henderson J, et al Cohort Profile: The 'Children of the 90s'—the index offspring of the Avon Longitudinal Study of Parents and Children. Int J Epidemiol 2012 10.1093/ije/dys064 PMC3600618 22507743

[21] Goodman R, Ford T, Richards H, Gatward R, Meltzer H. The Development and Well-Being Assessment: description and initial validation of an integrated assessment of child and adolescent psychopathology. J Child Psychol Psyc 2000; 41(5): 645–655. 10946756

[22] Wechsler D, Golombok J, Rust J. Wechsler Intelligence Scale for Children Sidcup, United Kingdom: The Psychological Corporation; 1992.

[23] Goodman R. The Strengths and Difficulties Questionnaire: a research note. J Child Psychol Psyc 1997; 38(5): 581–586. 10.1111/j.1469-7610.1997.tb01545.x 9255702

[24] Brant R. Assessing proportionality in the proportional odds model for ordinal logistic regression. Biometrics 1990; 46(4): 1171–1178. 2085632

[25] Anderson CA. An update on the effects of playing violent video games. J Adoles 2004; 27(1): 113–122. 10.1016/j.adolescence.2003.10.009 15013264

[26] Odgers CL, Caspi A, Broadbent JM, Dickson N, Hancox RJ, Harrington H, et al Prediction of differential adult health burden by conduct problem subtypes in males. Arch Gen Psychiat 2007; 64(4): 476–484. 10.1001/archpsyc.64.4.476 17404124

[27] Hasan Y, Begue L, Scharkow M, Bushman BJ. The more you play, the more aggressive you become: A long-term experimental study of cumulative violent video game effects on hostile expectations and aggressive behavior. J Exp Soc Psychol 2013; 49(2): 224–227.

[28] Parkes A, Sweeting H, Wight D, Henderson M. Do television and electronic games predict children's psychosocial adjustment? Longitudinal research using the UK Millennium Cohort Study. Arch Dis Child 2013; 98(5): 341–348. 10.1136/Archdischild-2011-301508 23529828 PMC3625829

[29] Engelhardt C. R., Mazurek M., Hilgard J., Bartholow B. D. (2015). Effects of violent-video-game exposure on aggressive behavior, aggressive-thought accessibility, and aggressive affect among adults with and without autism spectrum disorder. Psychological Science. 10.1177/0956797615583038 26113064

[30] Primack BA, Swanier B, Georgiopoulos AM, Land SR, Fine MJ. Association between media use in adolescence and depression in young adulthood: a longitudinal study. Arch Gen Psychiat 2009; 66(2): 181–188. 10.1001/archgenpsychiatry.2008.532 19188540 PMC3004674

